# Sex specific differences in HIV status disclosure and care engagement among people living with HIV in rural communities in Kenya and Uganda

**DOI:** 10.1371/journal.pgph.0000556

**Published:** 2023-04-07

**Authors:** Chinomnso N. Okorie, Sarah A. Gutin, Monica Getahun, Sarah A. Lebu, Jaffer Okiring, Torsten B. Neilands, Sarah Ssali, Craig R. Cohen, Irene Maeri, Patrick Eyul, Elizabeth A. Bukusi, Edwin D. Charlebois, Carol S. Camlin

**Affiliations:** 1 Department of Obstetrics, Gynecology & Reproductive Sciences, University of California, San Francisco, California, United States of America; 2 Department of Medicine, Division of Prevention Science, University of California, San Francisco, California, United States of America; 3 Infectious Diseases Research Collaboration, Kampala, Uganda; 4 School of Women and Gender Studies, Makerere University, Kampala, Uganda; 5 Kenya Medical Research Institute, Centre for Microbiology Research, Nairobi, Kenya; Stellenbosch University, SOUTH AFRICA

## Abstract

Non-disclosure of human immunodeficiency virus (HIV) status can hinder optimal health outcomes for people living with HIV (PLHIV). We sought to explore experiences with and correlates of disclosure among PLHIV participating in a study of population mobility. Survey data were collected from 1081 PLHIV from 2015–16 in 12 communities in Kenya and Uganda participating in a test-and-treat trial (SEARCH, NCT#01864603). Pooled and sex-stratified multiple logistic regression models examined associations of disclosure with risk behaviors controlling for covariates and community clustering. At baseline, 91.0% (n = 984) of PLHIV had disclosed their serostatus. Amongst those who had never disclosed, 31% feared abandonment (47.4% men vs. 15.0% women; *p* = 0.005). Non-disclosure was associated with no condom use in the past 6 months (aOR = 2.44; 95%CI, 1.40–4.25) and with lower odds of receiving care (aOR = 0.8; 95%CI, 0.04–0.17). Unmarried versus married men had higher odds of non- disclosure (aOR = 4.65, 95%CI, 1.32–16.35) and no condom use in the past 6 months (aOR = 4.80, 95%CI, 1.74–13.20), as well as lower odds of receiving HIV care (aOR = 0.15; 95%CI, 0.04–50 0.49). Unmarried versus married women had higher odds of non-disclosure (aOR = 3.14, 95%CI, 1.47–6.73) and lower odds of receiving HIV care if they had never disclosed (aOR = 0.05, 95%CI, 0.02–0.14). Findings highlight gender differences in barriers to HIV disclosure, use of condoms, and engagement in HIV care. Interventions focused on differing disclosure support needs for women and men are needed and may help facilitate better care engagement for men and women and improve condom use in men.

## Introduction

Disclosure of one’s HIV status has been shown to improve health outcomes among people living with HIV (PLHIV) [1,2] and can reduce human immunodeficiency virus (HIV) transmission by facilitating engagement in HIV-prevention and care [1,3–7]. Alternatively, non-disclosure, which is often a manifestation of HIV-related stigma (whether internalized, anticipated, or in response to enacted stigma), can negatively impact care outcomes [8–10].

HIV disclosure can be both a negative (harmful, stressful, unhelpful) and positive (supportive, and empowering) experience [11]. Positive disclosure experiences are linked to increased social support [12,13], reduced internalized stigma [9], improved mental health [9], and safer sexual behavior practices [14]. Alternatively, it can also be negative, leading to increased stigma, anxiety [2], violence or abuse in relationships [7,11,15–18], abandonment [9,13,19,20], fear of abandonment [13,21], discrimination [2,13,20], and rejection [2,20]. These issues are compounded by limited access to disclosure support, including a lack of feasible disclosure strategies and limited social and peer support, which impedes the benefits of HIV-status disclosure [2,3,13,19,20].

Reasons for PLHIV disclosure or non-disclosure can depend on social relationships, fear of disclosing, and concerns about stigma [11,19]. Disclosure can be instrumental to receiving support (financial, material, moral and emotional, treatment) for those who disclose. Disclosure can be used to explain a change in behavior or appearance and to promote HIV prevention or protect others from HIV. For those who choose not to disclose, it is a way to protect their identity and avoid stigma and discrimination while maintaining a sense of self and safety [8,11]. Some may disclose to avoid involuntary or second-hand disclosure [8]. However, this study focuses on voluntary disclosure (full, selective, or non-disclosure) [8,11]. In addition, PLHIV may disclose to various types of people for various reasons. A study in Uganda found that although a majority of PLHIV reported having disclosed their status, there were significant variations in persons to whom PLHIV disclosed (84% disclosed to family members, 63% to friends, 21% to workplace colleagues, and 18% to others) [8,19].

Further, disclosure is a highly gendered experience, with wide variations in experiences among men and women. Women have often experienced difficulty disclosing their HIV status to intimate partners, fearing negative reactions including violence, blame, and abandonment [2,6,9,19]. While men also fear negative consequences of disclosure including marital conflict, blame, being labeled as promiscuous, and abandonment by their partners, men have generally experienced less severe consequences and benefitted from more social support for disclosure, compared to women [1,18–23]. Further, men have been more likely to disclose to fellow men than women are to other women [3]. Studies in sub-Saharan Africa have also found that men use multiple strategies to avoid disclosure including introducing condom use under the pretext of family planning [2,24–27]. In addition, men often have more freedom compared to women to seek care in remote facilities outside of their community, thereby avoiding disclosure to their partners and families [22].

HIV-status disclosure remains a complex and challenging decision-making process for PLHIV [28–30]. Sex differences in decision-making processes surrounding the choice of individuals to whom to disclose, reasons for non-disclosure, and the effects of these on care engagement are poorly understood, particularly across typologies of relationship types (whether monogamous or concurrent). We sought to explore experiences with and correlates of disclosure among PLHIV participating in a study of population mobility in rural communities in Kenya and Uganda. Findings can be used to document the challenges faced by PLHIV and inform programs designed to reduce stigma and improve health outcomes.

## Materials and methods

### Study design and participants

**Fig 1:**
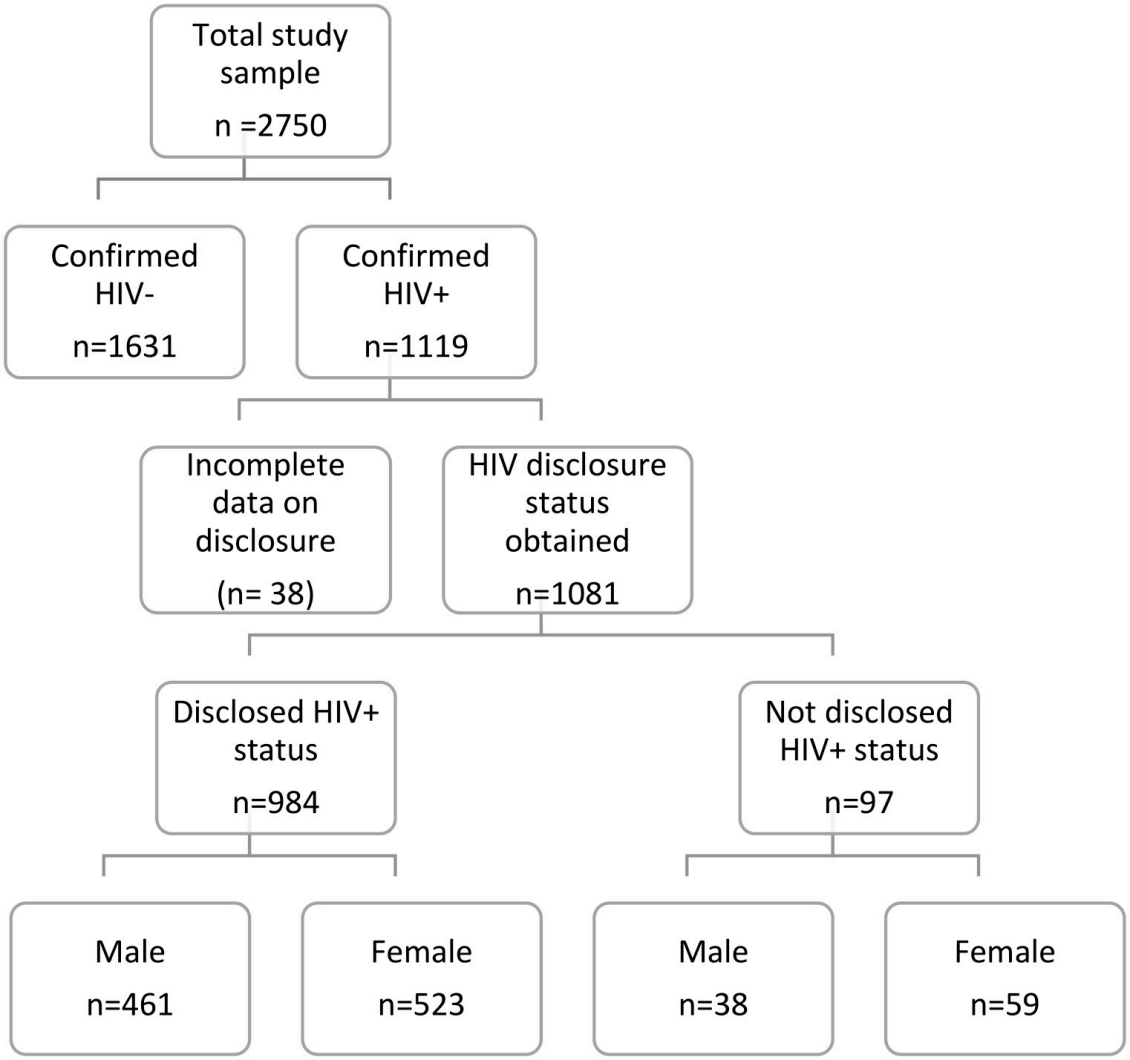
Analysis flowchart: Study participants’ HIV status, and whether disclosed status, at baseline. A diagram of study participants by HIV status and HIV status disclosure. In a sample of 2750 study paricipants (selected for balance by region, sex, mobility, and HIV status), 1119 were confirmed to be living with HIV, with 91.0% (n = 984) having ever disclosed their HIV status to others (outside a clinic setting) and 9.0% (n = 97) with HIV status not disclosed, at study baseline.

The *Understanding Mobility and Risk in SEARCH Communities* (R01MH104132) study [31,32] examined mobility, sexual behavior and HIV outcomes in a longitudinal cohort of 2,750 adults in 12 communities participating in a large-scale test-and-treat trial, the Sustainable East Africa Research in Community Health (SEARCH) study (NCT# 01864603), in Kenya and Uganda [29,33]. Methods are described in detail elsewhere [31]; in summary, a stratified random sampling design was used to select the sample of ∼200 individuals from each of 12 SEARCH communities, composed of eight roughly equally-sized groups of sex-specific, HIV-positive and HIV-negative, mobile (away from household six months or more in past 12 months and fewer than half of nights spent in household in past four months) and residentially stable (non-mobile), men and women. HIV-positive individuals and mobile individuals were oversampled to achieve the desired sample size in each stratum. This analysis uses baseline survey data collected from 1081 PLHIV for whom information on HIV status disclosure were available (Fig 1). HIV status disclosure was defined as PLHIV who reported voluntary disclosure of their HIV-positive status to at least one person.

### Procedures

An interviewer-administered survey was used to collect information on household residence(s) and composition, income and livelihoods, histories of migration during childhood and adulthood, patterns of mobility in the past six months (including destinations, reasons, duration and frequency of trips), sexual behavior (including sexual partnership histories over the past five years, using a calendar approach adapted from prior research studies [31,34]), and among PLHIV only, experiences with HIV stigma, disclosure, and engagement in HIV care and treatment. A detailed relationship history calendar permitted measurement of patterns and frequency of condom use, sexual partnership concurrency, and higher-risk partnerships (i.e. any report of a casual partner, commercial sex worker/client, one-night stand, or inherited partner (referring to the Luo practice of widow “inheritance” in which a widow and her children are retained in the family/lineage of her deceased husband; cultural practices include sexual contact with the inheritor [35]), over defined time-periods. Mobility was defined as any overnight travel in the past 6 months.

### Ethics statement

Ethical approvals were received from the University of California San Francisco Committee on Human Research (14–15058), Ethical Review Committee of the Kenya Medical Research Institute (KEMRI/SERU/CMR/3052), Makerere University School of Medicine Research and Ethics Committee (2015–040), and Uganda National Council for Science and Technology (HS 1834). All participants provided written informed consent before taking part in the study.

### Data collection

Data collection was conducted from February 2015-November 2016 by trained research assistants. Surveys were originally developed in English and then translated and administered (using portable tablets) in the local languages (Lusoga, Lugwere, Ateso, Runyankole, and Dholuo) of the research participants, following procedures for the protection of privacy and confidentiality. Surveys took 30 to 90 minutes to complete, were administered in a private area, and participants and interviewers were gender-matched to maximize comfort with sharing sensitive information. Participants were compensated the equivalent of $5 USD for their time and/or transport reimbursement, which is standard procedure in the region.

### Data analyses

Of the confirmed PLHIV at baseline (n = 1119), complete data for this analysis were available for 1081 participants (499 men, 582 women) (Fig 1). Descriptive statistics were computed to explore HIV status disclosure patterns by sex. Bivariate comparisons that accounted for clustering of individuals within communities (Rao-Scott F-tests) were used to characterize the relationship between HIV-positive status disclosure and sex. Pooled, and sex-stratified mixed effects logistic regression models were fitted to examine associations of HIV-positive status non-disclosure with sexual risk behaviors including past 6-month condom use, past year relationship concurrency, and HIV care engagement controlling for age, marital status, region and adjusted for clustering at the community-level. These factors were included in models because prior research has suggested an association between disclosure and age and marital status [36–38]. The sample in multivariable models was smaller (n = 906) because of case-wise deletion for missing data. All analyses were conducted using Stata statistical software version 16.1 (College Station, TX, USA).

## Results

### Characteristics of the sample

Of 1081 PLHIV included in the baseline analyses, 91.0% (n = 984; 523 female, 461 male) reported disclosing their status to at least one person and 9.0% (n = 97; n = 59 female, n = 38 male) reported non-disclosure of their HIV status to anyone (other than their healthcare provider) (Table 1). Overall, the majority of respondents (73.1%) were married, had received some primary-level education (83.2%), and were involved in low HIV-risk occupations (79.1%).

**Table 1:** Characteristics of adults who had and had not disclosed HIV-positive status at baseline, adjusted for community clustering.

Characteristics	Overall (n = 1081)		Non- Disclosed (n = 97)		Disclosed (n = 984)		F (df)	*p*
	n	%	n	%	n	%		
**Demographics**								
***Age (Mean*, *SE)***	42.2	0.47	40.1	1.06	42.4	0.55	F(1, 11) = 3.00	0.111
***Sex***							F(1, 11) = 1.837	0.202
Male	499	46.2	38	39.2	461	46.9		
Female	582	53.8	59	60.8	523	53.2		
***Occupational-Risk categories***							F(1.69,18.62) = 0.959	0.388
Low risk	855	79.1	72	78.3	783	82.6		
High risk	184	17.0	19	20.7	165	17.4		
***Educational Attainment***							F(1.95, 21.41) = 0.575	0.567
No School/missing	144	13.7	11	11.6	133	13.9		
Some primary/up to completed	874	83.2	82	86.3	792	82.9		
Some secondary or beyond	33	3.1	2	2.1	31	3.2		
***Marital Status***							F(1, 11) = 10.481	**0.008**
Currently married	789	73.1	57	58.8	732	74.5		
Other *	290	26.9	40	41.2	250	25.5		
**Migration history**								
***Any migration in past 5 years***							F(1, 11) = 1.228	0.292
No	412	38.1	41	42.3	371	37.7		
Yes	669	61.9	56	57.7	613	62.3		
***Any past 1 year migration***							F(1, 11) = 1.172	0.302
No	1002	92.7	93	95.9	909	92.4		
Yes	79	7.3	4	4.1	75	7.6		
***Any past 2 year migration***							F(1, 11) = 0.691	0.424
No	943	87.2	88	90.7	855	86.9		
Yes	138	12.8	9	9.3	129	13.1		
**Mobility patterns**								
***Any past 6 month work travel***							F(1, 11) = 0.913	0.360
No	937	86.7	87	89.7	850	86.4		
Yes	144	13.3	10	10.3	134	13.6		
***Any 6 past month non-work travel***							F(1, 11) = 2.337	0.155
No	595	55.0	60	61.9	535	54.4		
Yes	486	45.0	37	38.1	449	45.6		
***Any past 6 month (overnight) travel***							F(1, 11) = 1.365	0.267
Mobile	418	38.7	43	44.3	375	38.1		
Non-mobile	663	61.3	54	55.7	609	61.9		
**Sexual behavior**								
***Any condom use in past 6 months***							F(1, 11) = 8.605	**0.014**
Yes condom use	529	58.3	29	37.7	500	60.2		
Never	379	41.7	48	62.3	331	39.8		
***Any high-risk partnerships*, *2015–16*** **							F(1, 11) = 0.5626	0.469
No	923	85.4	81	83.5	842	85.6		
Yes	158	14.6	16	16.5	142	14.4		
***Any concurrent partnerships*, *past 6 months*** ***							F(1, 11) = 9.2789	**0.011**
No	922	79.2	92	9.9	830	78.0		
Yes	159	20.8	5	3.2	154	22.0		
**HIV care engagement**								
***Currently receiving HIV Care***							F(1, 11) = 42.580	**<0.001**
No	46	4.3	21	21.7	25	2.5		
Yes	1035	95.7	76	78.4	959	97.5		
***Ever enrolled in ART program***							F(1.55,17.01) = 31.509	**<0.001**
No	66	6.1	26	26.8	40	4.1		
Yes	1015	93.9	71	73.2	944	95.9		
***Currently taking ART***							F(1.60,17.55) = 23.927	**<0.001**
No	80	7.4	26	26.8	54	5.5		
Yes	1001	92.6	71	73.2	930	94.5		
***Attending clinic***							F(1, 11) = 0.0247	0.878
One clinic	986	97.5	71	97.3	915	97.6		
More than one clinic	25	2.5	2	2.7	23	2.5		
***Ever missed appointments/ Dropped out of care for a time***							F(1.81, 19.91) = 26.754	**<0.001**
No	928	89.7	69	90.8	859	89.6		
One or more appointments	107	10.3	7	1.0	100	10.4		

Bivariate comparisons that accounted for clustering of individuals within communities (Rao-Scott F-tests) were used to examine the relationship between selected characteristics and disclosure of HIV status, at baseline. Data are column percentages; percentages sum to greater than 100% because multiple responses were permitted.

*Marital status, “Other”: Single, widowed, divorced, separated, do not know, missing.

** Higher-risk partnerships: Any casual partner, commercial sex worker/client, one-night stand, and inherited partner.

*** Concurrent partnerships: Any overlapping sexual partners within any month in the period (2015–16).

### PLHIV who have disclosed their HIV status

The majority of those who reported ever disclosing their status were female (53.2%, n = 523) and 74.5% were currently married (Table 1). The mean age of those who had disclosed was 42 years. Further, 60.2% reported any past 6-month condom use and 22.0% reported being in concurrent relationships in 2015–16. Almost all of those who had disclosed (97.7%) were receiving HIV care, were enrolled in antiretroviral therapy (ART) programs (95.9%), were taking ART (94.5%), and were attending a clinic (97.6%). In addition, 38.1% (n = 375) of mobile PLHIV disclosed while 61.9% (n = 609) of non-mobile PLHIV had disclosed (Table 1).

**Fig 2:**
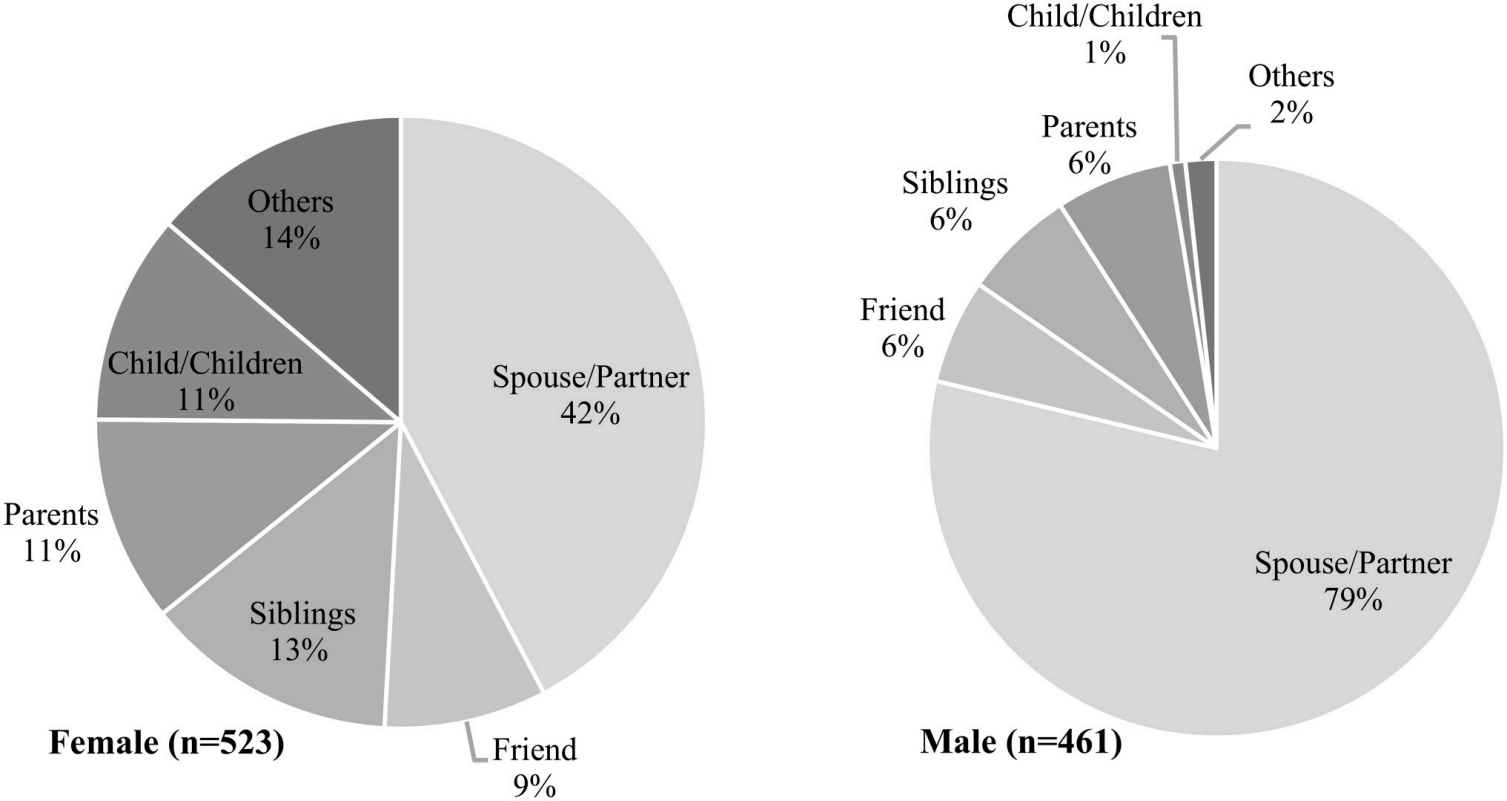
Types of individuals to whom PLHIV first disclosed their HIV status, by sex (n = 984). The graph shows the proportion (%) of individuals to whom PLHIV first disclosed their HIV status (n = 984, F(4.18, 45.94) = 20.7750, p<0.001). Data shown are coloumn percentages, by sex. Test statistic is adjusted for clustering at the community-level.

Among PLHIV who had disclosed, the first person to whom they had most commonly disclosed was a spouse/partner (59.4%) (Fig 2). When stratified by sex, differences were seen in whom men and women disclosed to. Overall, men were significantly more likely to disclose to a spouse/partner first, compared to women (78.7% vs. 42.0%, F(4.18, 45.94) = 20.7750, p<0.001) (Fig 2). However, even among those who reported disclosing their status to at least one person, 21.4% wished to disclose to others but felt they could not (Table 2). The majority of women wanted to disclose to a friend (25%), and/or mother (25%), while men preferred to disclose to a brother (33%), or friend (31%) (Table 2). The main reasons for not disclosing to additional people were fear of being judged (56.9%), fear of abandonment (27.0%), and other reasons (25.6%) (Table 2). The only significant differences observed between men and women were feeling guilty for extramarital affairs (15.5% men vs. 3.5% women, F(1, 11) = 5.985, *p* = 0.032), or other reasons including not trusting others and fears of hurting/stressing others (9.3% men vs. 39.5% women, F(1, 11) = 18.717, p = 0.001) (Table 2).

**Table 2:** Patterns of HIV disclosure among PLHIV who have already disclosed HIV status, by sex and adjusted for community clustering.

	**Overall**	**%**	**Female**	**%**	**Male**	**%**	**F (df)**	***p***
	(n = 984)		(n = 523)		(n = 461)			
**PLHIV wished to disclose to others to whom they had not yet disclosed**								
No	773	78.6	409	78.2	364	79.0	F(1, 11) = 0.024	0.879
Yes	211	21.4	114	21.8	97	21.0		
	**Overall**	%	**Female**	%	**Male**	%		***p***
	(n = 211)		(n = 114)		(n = 97)			
**Other people to whom PLHIV would like to disclose HIV status, but felt they could not** *								
Friend	59	28.0	29	25.4	30	30.9	F(1, 11) = 0.591	0.458
Mother	54	25.6	28	24.6	26	26.8	F(1, 11) = 0.357	0.563
Brother	44	20.9	12	10.5	32	33.0	F(1, 11) = 7.377	**0.020**
Sister	41	19.4	16	14.0	25	25.8	F(1, 11) = 2.615	0.134
Spouse/partner	35	16.6	23	20.2	12	12.4	F(1, 11) = 9.265	**0.011**
Other relatives	25	11.8	20	17.5	5	5.2	F(1, 11) = 4.241	0.064
Child/children	19	9.0	11	9.6	8	8.2	F(1, 11) = 0.117	0.739
Father	15	7.1	9	7.9	6	6.2	F(1, 11) = 0.672	0.430
Others	8	3.8	7	6.1	1	1.0	F(1, 11) = 3.835	0.076
Employer	4	1.9	0	0.0	4	4.1	F(1, 11) = 2.741	0.126
**Reasons for not disclosing further** *								
Afraid of being judged	120	56.9	49	43.0	71	73.2	F(1, 11) = 4.255	0.064
Afraid of being abandoned	57	27.0	27	23.7	30	30.9	F(1, 11) = 0.469	0.508
Afraid of violence	25	11.8	16	14.0	9	9.3	F(1, 11) = 0.938	0.354
Felt guilty (extramarital affair)	19	9.0	4	3.5	15	15.5	F(1, 11) = 5.985	**0.032**
Not disclosed for other reasons**	54	25.6	45	39.5	9	9.3	F(1, 11) = 18.717	**0.001**

Bivariate comparisons that accounted for clustering of individuals within communities (Rao-Scott F-tests) were used to examine associations of disclosure characteristics by sex; data are column percentages.

*Percentages sum to greater than 100% because multiple responses were permitted.

**Other reasons includes being distrustful of others, fear of hurting/stressing others, not found the right time to disclose, not interested in disclosing, those advised by health worker/others not to disclose, afraid of being ridiculed/made fun of, afraid of losing job, current partner discourages disclosure, and those not ready to disclose.

### PLHIV who have not disclosed their HIV status

Of the 9.0% (n = 97) of PLHIV who had not disclosed their status, the majority (60.8%) were female and reported being currently married (39.2%, n = 38) (Table 1). Further bivariate analyses among PLHIV who had not disclosed their status showed that 37.7% (n = 29) reported any past 6-month condom use and 3.2% reported being in concurrent relationships. HIV care engagement was lower among those who had not disclosed compared to those who had disclosed, with 78.4% receiving HIV care, 73.2% enrolled in ART programs and taking ART (n = 71 for each), and 97.3% attending one clinic (Table 1). In addition, of the participants who had not disclosed their HIV status, 44.3% were mobile compared to 55.7% who were non-mobile (Table 1). The main reasons for not disclosing HIV status included being afraid of being judged (54.6%) and afraid of being abandoned (30.9%) (Table 3). Significant differences were observed between men and women with men being more afraid of being abandoned than women (47.4% men vs. 15% women, F(1, 11) = 12.440, p = 0.005), and more men (27.5%) than women (15.4%) having other reasons (e.g. not being interested in disclosing (30.8%) (F(1, 11) = 13.452, p = 0.004) (Table 3).

**Table 3:** Reasons for non-disclosure of HIV status, by sex and adjusted for community clustering.

**Reasons for non-disclosure** *	**Overall**	**%**	**Female**	**%**	**Male**	**%**	**F (df)**	***p***
	(n = 97)		(n = 59)		(n = 38)			
Afraid of being judged	53	54.6	29	36.3	24	63.2	F(1, 11) = 0.569	0.467
Afraid of being abandoned	30	30.9	12	15.0	18	47.4	F(1, 11) = 12.440	**0.005**
Afraid of violence	17	17.5	13	16.3	4	10.5	F(1, 11) = 1.196	0.297
Felt guilty	8	8.2	4	5.0	4	10.5	F(1, 11) = 0.504	0.493
Afraid for other reasons	26	26.8	22	27.5	4	15.4	F(1, 11) = 13.452	**0.004**
**Other Reasons for non-disclosing (specified)**	**Overall**	**%**	**Female**	**%**	**Male**	**%**	**F (df)**	***p***
	(n = 97)		(n = 59)		(n = 38)			
Denial/Non Acceptance	6	23.1	6	27.3	0	0.0	F(2.41, 19.30) = 0.630	0.572
Fear Of Hurting/Stress	4	15.4	3	13.6	1	25.0		
Not Interested In Disclosure	8	30.8	7	31.8	1	25.0		
Other**	8	30.8	6	27.3	2	50.0		

Bivariate comparisons that accounted for clustering of individuals within communities (Rao-Scott F-tests) were used to examine reasons for not disclosing HIV status by sex; data are row percentages. Question stem for PLHIV who have not disclosed (n = 97): “Can you tell the reason why you haven’t felt able to disclose your status to anyone?”, 108 PLHIV responded to one or more reasons for not disclosing status (58 females and 50 males).

*percentages sum to greater than 100% because multiple responses were permitted.

**Other includes Distrusting of Others, No one to disclose to, Afraid of losing job.

### Analysis of HIV non-disclosure patterns

Multivariable analyses using sex-pooled and sex stratified logistic regression models revealed that compared to those who were married, those who were unmarried had three times the odds of not disclosing an HIV-positive status (aOR = 3.17; 95%CI, 1.69–5.94, p<0.001). PLHIV reporting no past 6-month condom use had over two times the odds of non-disclosure relative to those reporting any condom use during the period (aOR = 2.44; 95%CI, 1.40–4.25, p = 0.002). Those reporting concurrent partnerships also had lower odds of non-disclosure compared to those in monogamous relationships (aOR = 0.37; 95%CI, 0.14–0.99, p = 0.047). Furthermore, those currently receiving HIV care had lower odds of non-disclosure relative to those not receiving care (aOR = 0.08; 95%CI, 0.04–0.17, p<0.001).

In sex-stratified models, men who were unmarried compared to married had almost five times the odds of not disclosing an HIV-positive status (aOR = 4.65; 95%CI, 1.32–16.35, p = 0.017). Also, men with no past 6-month condom use had almost five times the odds of non-disclosure compared to those who reported any condom use (aOR = 4.80; 95%CI, 1.74–13.20, p = 0.002) and men receiving HIV care compared to men not in care had lower odds of non-disclosure (aOR = 0.15; 95% CI, 0.04–0.49, p = 0.002). Women who were unmarried compared to married had three times the odds of not disclosing an HIV-positive status (aOR = 3.14; 95%CI, 1.47–6.73, p = 0.003). In addition, women receiving HIV care compared to those not in care had 95% lower odds of non-disclosure (aOR = 0.05; 95%CI, 0.02–0.14, p<0.001) (Table 4).

**Table 4:** Multivariate analysis examining factors associated with non-disclosure at basline, straified by sex and adjusted for community clustering.

	TOTAL n = 906				MEN n = 471				WOMEN n = 435			
	Unadjusted Models *				Adjusted Models				Adjusted Models			
	OR	95% CI		*p*	aOR	95% CI		*p*	aOR	95% CI		*p*
**Age (continuous)**	0.98	0.96	1.00	0.107	1.00	0.97	1.04	0.890	0.96	0.92	0.99	**0.017**
**Region**												
Kenya	REF	-	-	-	-	-	-	-	-	-	-	-
Uganda E	1.82	0.89	3.70	0.099	2.09	0.72	6.02	0.173	1.46	0.52	4.13	0.472
Uganda SW	1.71	0.92	3.18	0.088	1.78	0.63	5.02	0.274	1.60	0.72	3.52	0.248
**Sex**												
Male	REF	-	-	-	-	-	-	-	-	-	-	-
Female	1.25	0.71	2.20	0.441	-	-	-	-	-	-	-	-
**Marital status**												
Currently married	REF	-	-	-	REF	-	-	-	REF	-	-	-
*Other	3.17	1.69	5.94	**<0.001**	4.65	1.32	16.35	**0.017**	3.14	1.47	6.73	**0.003**
**Any condom use in last 6 months**												
Yes condom use	REF	-	-	-	REF	-	-	-	REF	-	-	-
Never	2.44	1.40	4.25	**0.002**	4.80	1.74	13.20	**0.002**	1.81	0.89	3.70	0.104
**Any Concurrent Partnership 2015–16**												
No	REF	-	-	-	REF	-	-	-	REF	-	-	-
Yes	0.37	0.14	0.99	**0.047**	0.53	0.17	1.65	0.276	0.14	0.02	1.22	0.075
**Receiving HIV care**												
No	REF	-	-	-	REF	-	-	-	REF	-	-	-
Yes	0.08	0.04	0.17	**<0.001**	0.15	0.04	0.49	**0.002**	0.05	0.02	0.14	**<0.001**

Multivariate Analysis using mixed effect logistic regression to measure non-disclosure, straified by sex at baseline and adjusted for community clustering.

*Marital status-other = (single, widowed, divorced, separated, do not know, missing).

## Discussion

This study highlights the impact of sex-specific patterns of HIV-disclosure amongst a cohort of PLHIV in the context of rapid ART expansion in rural Kenya and Uganda. At baseline, the majority of PLHIV had disclosed their status to at least one person, predominently a spouse/partner. Even among those who had disclosed, both men and women expressed the desire to further disclose their status to a close friend or family member. Those who had never disclosed had higher odds of being unmarried and never using condoms and lower odds of HIV care engagement. When stratified by sex, we found that men who have never disclosed have higher odds of being unmarried and reporting no condom-use in the past 6-months and lower odds of HIV care engagement, while women who had never disclosed their HIV status had higher odds of being unmarried and lower odds of HIV care engagement.

With the aid of community researchers and meticulous community engagement in rural Kenya and Uganda, this study was able to attain an HIV-status disclosure rate over 90% at baseline. This finding aligns with other studies conducted in Cameroon, Nigeria, Malawi, and Zimbabwe that have reported disclosure rates above 80% [29,39–44]. Even with high disclosure rates in this study, disclosing was not without its emotional, relational, and psychological hardships [45]. Among PLHIV who had already disclosed their status, we found that many wished to disclose to others, but feared judgment and/or felt guilty for extramarital affairs. Research has established that there are negative consequences of disclosing, including stigma, anxiety, fear of abandonment, discrimination, rejection, and unhealthy relationships with others [2,3,13,19,20]. Therefore, a lack of access to suitable disclosure strategies and a lack of peer/social support can continue to limit HIV status disclosure and an individual’s well-being.

In this study, the majority of PLHIV had disclosed to at least one person, most commonly a spouse/partner. In addition, we saw some differences by sex, with men overwhelmingly disclosing to their spouse/partner (79% compared to 42% for women). It is possible that men disclose to partners/spouses more than women because they feel greater social support from their female parters. Social support is an important aspect of psychological adjustment that can promote well-being for many PLHIV [46,47] and is an essential resource for coping [46]. However, social support varies by sex. Men who disclose tend to benefit from increased social support [22] and the consequences of disclosure are less severe [2,23]. However, amongst young women, disclosure can be more difficult as many fear negative reactions including upsetting a partner, violence, abandonment, and blame [2,19,41]. Therefore, partner notification policies and support programs must be responsive to the potential negative consequences associated with disclosure for women [48]. This highlights the need, particularly for women, for facilitated couples disclosure. In addition, even though men benefit more than women from increased social support following disclosure of their HIV-status [22], there is a need for male-centered interventions in HIV care [42], because men have reported increased care and support when they disclose to fellow men compared to when women disclose to women [3]. Another possible next step is to create interventions and treatments that support the family unit (mother, father, and child) as a focal point to increase HIV disclosure and care engagement and to motivate more HIV disclosure to spouses, particularly among women, either before or after initiating ART.

The risks of disclosure are particularly salient for women. More women living with HIV in this study who had not disclosed their status reported fears of violence and judgement as their primary concern for non-disclosure whereas men reported being afraid of abandonment and judgment. This coincides with qualitative research from Kenya and Uganda which has found that negative consequences of disclosure, including severe consequences (i.e. violence), were reported disproportionately by women [2]. In that study, women expressed anxieties around partner abandonment or violence and perceived greater HIV/AIDS stigma [39], whereas men were concerned about their partners perceiving them as promiscuous [2,14,16,39]. Disclosure approaches within relationships are needed as successful disclosure within intimate partnerships can lead to engagement in risk-reduction strategies [2].

Sex-specific factors associated with HIV disclosure patterns need interventions to go beyond a majority of PLHIV reporting disclosure to a spouse/partner. This includes creating a safe space for both sexes to equally express their status and further disclose to others within their family/extended family. Expanding support for assisted disclosure for couples and families is critical and efforts to strengthen health systems capacity for clinician or counselor-assisted disclosure is needed. Yet such strategies should be gender-sensitive and attuned to men’s and women’s differing needs and experiences. For example, interventions to encourage and support women in safely disclosing their status can focus on restructuring comprehensive support services and re-training peer educators–a crucial support element in creating a safe disclosure environment for women.

Furthermore, factors associated with non-disclosure vary by sex and include marital status, condom use, and engagement with HIV care. Unmarried men (single, divorced or widowed) had five times the odds of non-disclosure compared to those who were married. Those who are married do not have to contend with the same level of fear as those who are unmarried. In addition, men who had not disclosed were not using condoms. This effect was not seen among women. It is possible that men feared that using condoms might signal their HIV-positive status to a partner or lead to stigma, as prior literature among men who have sex with men has suggested [49]. Research suggests that sometimes men have used avoidant disclosure strategies (i.e. introduction of condom use) under the pretext of family planning and protecting their partners while women have used such techniques when they were unsure of their partner’s HIV status [2]. it is usually easier for men living with HIV than women to disclose based on gender differences in sexual decision-making power [2]. Women have reported substantial difficulties in negotiating condom use and may not culturally regard using a condom as a sense of empowerment and control over their own bodies [15]. This finding suggests the importance of disclosure to ensure optimal risk-reduction techniques such as condom use and HIV care engagement.

In addition, among both women and men, the odds of non-disclosure were reduced among those receiving HIV care. HIV status non-disclosure can play a critical role in care disruption resulting in the inability or reluctance to take medications or attend clinic, for fear of disclosing one’s status [8,19]. The odds of non-disclosure were lower for men than for women who were engaged in care. This can be linked to existing evidence that found that men were often enrolled in HIV care secretively or at distant clinics while women found challenges initiating or staying engaged in care [2].

Prior research has highlighted that mobility is a highly gendered experience that is associated with higher-risk sexual behaviors [50]. Migrants who are more mobile engage in higher-risk sexual behaviors while travelling, increasing their risk of post-migration HIV-acquisition [51]. In this study, neither mobility or migration affected HIV disclosure rates at baseline. Nevertheless, HIV disclosure responses were different for men and women.

### Limitations

The cross-sectional nature of the study limits our ability to draw causal inferences. All community participants in this study were PLHIV attending clinical care in communities undergoing rapid ART scale up as a part of a community-based intervention study, thus limiting generalizability; however, study contexts are illustrative of similar communities in high HIV prevalence regions with varied rates of disclosure, mobility, access to care, and sex-specific disclosure issues.

## Conclusion

This study highlights the substantial gender differences and barriers to HIV disclosure. The findings demonstrate a need for attention to the differing disclosure experiences and support needs for both women and men in East Africa despite mobility. As the experience of this test-and-treat trial demonstrates, the rapid scale-up of HIV testing and ART rollout makes a critical mass of individuals newly aware of their HIV diagnosis, and newly presented with the dilemmas of disclosure, rendering the need for a robust programmatic response all the more urgent. However, increasing the number of people who have disclosed is only possible in a conducive environment. Governments and AIDS organizations must refocus and reform programs/services to provide adequate emotional and optimal organizational support to those who disclose, including peer support, counseling, and providing adequate training for health workers to offer proper HIV care and counseling [5]. Creating a safe space for disclosure, particularly for women disclosing to partners, is an area of priority within the context of rapid ART expansion.

## Supporting information

S1 Text Inclusivity questionnaire: PLOS questionnaire on inclusivity in global research. (DOCX)

## References

[1] Norman A, Chopra M, Kadiyala S. Factors Related to HIV Disclosure in 2 South African Communities. Am J Public Health. 2007 Oct;97(10):1775–81. doi: 10.2105/AJPH.2005.082511 17761582 PMC1994182

[2] Maeri I, El Ayadi A, Getahun M, Charlebois E, Akatukwasa C, Tumwebaze D, et al. “How can I tell?” Consequences of HIV status disclosure among couples in eastern African communities in the context of an ongoing HIV “test-and-treat” trial. AIDS Care. 2016 Jun 2;28(Suppl 3):59–66. doi: 10.1080/09540121.2016.1168917 27421052 PMC5751752

[3] Atuyambe LM, Ssegujja E, Ssali S, Tumwine C, Nekesa N, Nannungi A, et al. HIV/AIDS status disclosure increases support, behavioural change and, HIV prevention in the long term: A case for an Urban Clinic, Kampala, Uganda. BMC Health Serv Res. 2014;14:1–11. 24950958 10.1186/1472-6963-14-276 PMC4076501

[4] Chamie G, Clark TD, Kabami J, Kadede K, Ssemmondo E, Steinfeld R, et al. A hybrid mobile HIV testing approach for population-wide HIV testing in rural East Africa: an observational study. Lancet HIV. 2016 Mar 1;3(3):e111–9. 26939734 10.1016/S2352-3018(15)00251-9 PMC4780220

[5] Paxton S. The paradox of public HIV disclosure. AIDS Care. 2002 Aug 1;14(4):559–67. doi: 10.1080/09540120208629674 12204158

[6] Sharma M, Barnabas RV, Celum C. Community-based strategies to strengthen men’s engagement in the HIV care cascade in sub-Saharan Africa. PLoS Medicine. 2017 Apr 11;14(4):e1002262. doi: 10.1371/journal.pmed.1002262 28399122 PMC5388461

[7] Tam M, Amzel A, Phelps BR. Disclosure of HIV serostatus among pregnant and postpartum women in sub-Saharan Africa: a systematic review. AIDS Care. 2015 Apr 3;27(4):436–50. doi: 10.1080/09540121.2014.997662 25636060

[8] Obermeyer CM, Baijal P, Pegurri E. Facilitating HIV Disclosure Across Diverse Settings: A Review. Am J Public Health. 2011 Jun;101(6):1011–23. doi: 10.2105/AJPH.2010.300102 21493947 PMC3093267

[9] Geary C, Parker W, Rogers S, Haney E, Njihia C, Haile A, et al. Gender differences in HIV disclosure, stigma, and perceptions of health. AIDS Care. 2014 Nov 2;26(11):1419–25. doi: 10.1080/09540121.2014.921278 24921155

[10] Earnshaw VA, Smith LR, Shuper PA, Fisher WA, Cornman DH, Fisher JD. HIV stigma and unprotected sex among PLWH in KwaZulu-Natal, South Africa: a longitudinal exploration of mediating mechanisms. AIDS Care. 2014 Dec 2;26(12):1506–13. doi: 10.1080/09540121.2014.938015 25040218 PMC4188743

[11] ARCH HIV/AIDS Resources and Community [Internet]. [cited 2021 Jul 26]. https://www.archguelph.ca/sites/default/files/HIV%20Disclosure%20Guide.pdf.

[12] Maman S, Van Rooyen H, Groves AK. HIV status disclosure to families for social support in South Africa (NIMH Project Accept/HPTN 043). AIDS Care. 2014 Feb 1;26(2):226–32. doi: 10.1080/09540121.2013.819400 23875539 PMC4074900

[13] Ogoina D, Ikuabe P, Ebuenyi I, Harry T, Inatimi O, Chukwueke O. Types and predictors of partner reactions to HIV status disclosure among HIV infected adult nigerians in a tertiary hospital in the Niger delta. Afr Health Sci. 2015 Mar 9;15(1):10–8. doi: 10.4314/ahs.v15i1.2 25834525 PMC4370137

[14] Rujumba J, Neema S, Byamugisha R, Tylleskär T, Tumwine JK, Heggenhougen HK. “Telling my husband I have HIV is too heavy to come out of my mouth”: Pregnant women’s disclosure experiences and support needs following antenatal HIV testing in eastern Uganda. J Int AIDS Soc. 2012 Apr;15(2):17429. doi: 10.7448/ias.15.2.17429 22905360 PMC3494159

[15] Jewkes R, Morrell R. Gender and sexuality: emerging perspectives from the heterosexual epidemic in South Africa and implications for HIV risk and prevention. J Int AIDS Soc. 2010 Dec;13:1–1. 20181124 10.1186/1758-2652-13-6 PMC2828994

[16] Stangl AL, Earnshaw VA, Logie CH, van Brakel W, Simbayi L C., Barré I, et al. The Health Stigma and Discrimination Framework: a global, crosscutting framework to inform research, intervention development, and policy on health-related stigmas. BMC Med. 2019 Dec;17:1–3. 30764826 10.1186/s12916-019-1271-3 PMC6376797

[17] Shamu S, Zarowsky C, Shefer T, Temmerman M, Abrahams N. Intimate partner violence after disclosure of HIV test results among pregnant women in Harare, Zimbabwe. PLoS ONE. 2014 Oct 28;9(10):e109447. doi: 10.1371/journal.pone.0109447 25350001 PMC4211664

[18] Dessalegn NG, Hailemichael RG, Shewa-amare A, Sawleshwarkar S, Lodebo B, Amberbir A, et al. HIV Disclosure: HIV-positive status disclosure to sexual partners among individuals receiving HIV care in Addis Ababa, Ethiopia. PloS One. 2019 Feb 15;14(2):e0211967. doi: 10.1371/journal.pone.0211967 30768642 PMC6415764

[19] Ssali SN, Atuyambe L, Tumwine C, Segujja E, Nekesa N, Nannungi A, et al. Reasons for disclosure of HIV status by people living with HIV/AIDS and in HIV care in Uganda: An exploratory study. AIDS Patient Care STDs. 2010 Oct 1;24(10):675–81. doi: 10.1089/apc.2010.0062 20863244 PMC3826576

[20] Dageid W, Govender K, Gordon SF. Masculinity and HIV disclosure among heterosexual South African men: Implications for HIV/AIDS intervention. Cult Health Sex. 2012 Sep 1;14(8):925–40. doi: 10.1080/13691058.2012.710337 22943462

[21] King R, Katuntu D, Lifshay J, Packel L, Batamwita R, Nakayiwa S, et al. Processes and outcomes of HIV serostatus disclosure to sexual partners among people living with HIV in Uganda. AIDS Behav. 2008;12:232–43. doi: 10.1007/s10461-007-9307-7 17828450

[22] Brown LB, Getahun M, Ayieko J, Kwarisiima D, Owaraganise A, Atukunda M, et al. Factors predictive of successful retention in care among HIV-infected men in a universal test-and-treat setting in Uganda and Kenya: A mixed methods analysis. PloS One. 2019 Jan 23;14(1):e0210126. doi: 10.1371/journal.pone.0210126 30673744 PMC6343966

[23] Deribe K, Woldemichael K, Njau BJ, Yakob B, Biadgilign S, Amberbir A. Gender differences regarding barriers and motivators of HIV status disclosure among HIV-positive service users. Sahara-J: Journal of Social Aspects of HIV/AIDS. 2010;7(1). doi: 10.1080/17290376.2010.9724953 21409293

[24] Deribe K, Woldemichael K, Wondafrash M, Haile A, Amberbir A. Disclosure experience and associated factors among HIV positive men and women clinical service users in southwest Ethiopia. BMC Public Health. 2008 Dec;8:1–0. 18312653 10.1186/1471-2458-8-81 PMC2275263

[25] Medley A, Garcia-Moreno C, McGill S, Maman S. Rates, barriers and outcomes of HIV serostatus disclosure among women in developing countries: Implications for prevention of mother-to-child transmission programmes. Bull World Health Organ. 2004;82:299–307. 15259260 PMC2585956

[26] Walcott MM, Hatcher AM, Kwena Z, Turan JM. Facilitating HIV status disclosure for pregnant women and partners in rural Kenya: a qualitative study. BMC Public Health. 2013 Dec;13(1):1–3. doi: 10.1186/1471-2458-13-1115 24294994 PMC3907031

[27] Jewkes R, Morrell R. Hegemonic Masculinity, Violence, and Gender Equality: Using Latent Class Analysis to Investigate the Origins and Correlates of Differences between Men. Men and Masculinities. 2018 Oct;21(4):547–71.

[28] Ayieko J, Brown L, Anthierens S, Van Rie A, Getahun M, Charlebois ED, et al. “Hurdles on the path to 90-90-90 and beyond”: Qualitative analysis of barriers to engagement in HIV care among individuals in rural East Africa in the context of test-and-treat. PloS One. 2018 Aug 30;13(8):e0202990. doi: 10.1371/journal.pone.0202990 30161172 PMC6116983

[29] Chamie G, Kamya MR, Petersen ML, Havlir DV. Reaching 90–90–90 in rural communities in East Africa: lessons from the Sustainable East Africa Research in Community Health Trial. Curr Opin HIV AIDS. 2019 Nov;14(6):449. doi: 10.1097/COH.0000000000000585 31589172 PMC6798741

[30] UN Joint Programme on HIV/AIDS (UNAIDS). The Gap Report. 2014. https://www.refworld.org/docid/53f1e1604.html

[31] Camlin CS, Akullian A, Neilands TB, Getahun M, Bershteyn A, Ssali S, et al. Gendered dimensions of population mobility associated with HIV across three epidemics in rural Eastern Africa. Health & Place. 2019 May 1;57:339–51. doi: 10.1016/j.healthplace.2019.05.002 31152972 PMC6589115

[32] Camlin CS, Akullian A, Neilands TB, Getahun M, Eyul P, Maeri I, et al. Population mobility associated with higher risk sexual behaviour in eastern African communities participating in a Universal Testing and Treatment trial. J Int AIDS Soc. 2018 Jul;21:e25115. doi: 10.1002/jia2.25115 30027668 PMC6053476

[33] Havlir DV, Balzer LB, Charlebois ED, Clark TD, Kwarisiima D, Ayieko J, et al. HIV Testing and Treatment with the Use of a Community Health Approach in Rural Africa. N Engl J Med. 2019 Jul 18;381(3):219–29. doi: 10.1056/NEJMoa1809866 31314966 PMC6748325

[34] Luke N, Clark S, Zulu EM. The relationship history calendar: improving the scope and quality of data on youth sexual behavior. Demography. 2011 Aug 1;48(3):1151–76. doi: 10.1007/s13524-011-0051-2 21732169 PMC3354771

[35] Camlin CS, Kwena ZA, Dworkin SL, Cohen CR, Bukusi EA. “She mixes her business”: HIV transmission and acquisition risks among female migrants in western Kenya. Social Science & Medicine. 2014 Feb 1;102:146–56. doi: 10.1016/j.socscimed.2013.11.004 24565152 PMC3935174

[36] Dankoli RS, Aliyu AA, Nsubuga P, Nguku P, Ossai OP, Tukur D, et al. HIV disclosure status and factors among adult HIV positive patients in a secondary health facility in North-Eastern Nigeria, 2011. Pan Afr Med J. 2014;18(Suppl 1). 10.11694/pamj.supp.2014.18.1.3551 PMC4199349 25328623

[37] Amoran OE. Predictors of disclosure of sero-status to sexual partners among people living with HIV/AIDS in Ogun State, Nigeria. Niger J Clin Pract. 2012;15(4):385–90. doi: 10.4103/1119-3077.104507 23238184

[38] Simbayi L, Zungu N, Evans M, Mehlomakulu V, Kupamupindi T, Mafoko G, et al. HIV Serostatus Disclosure to Sexual Partners Among Sexually Active People Living with HIV in South Africa: Results from the 2012 National Population-Based Household Survey. AIDS Behav. 2017 Jan;21:82–92. doi: 10.1007/s10461-015-1278-5 26767538

[39] Anglewicz P, Chintsanya J. Disclosure of HIV status between spouses in rural Malawi. AIDS Care. 2011 Aug 1;23(8):998–1005. doi: 10.1080/09540121.2010.542130 21390889 PMC3371657

[40] Olagbuji BN, Ezeanochie MC, Agholor KN, Olagbuji YW, Ande AB, Okonofua FE. Spousal disclosure of HIV serostatus among women attending antenatal care in urban Nigeria. J Obstet Gynaecol. 2011 Aug 1;31(6):486–8. doi: 10.3109/01443615.2011.563637 21823844

[41] Patel R, Ratner J, Gore-Felton C, Kadzirange G, Woelk G, Katzenstein D. HIV disclosure patterns, predictors, and psychosocial correlates among HIV-positive women in Zimbabwe. AIDS Care. 2012 Mar 1;24(3):358–68. doi: 10.1080/09540121.2011.608786 21902570 PMC3243809

[42] Kusemererwa S, Akena D, Nakanjako D, Kigozi J, Nanyunja R, Nanfuka M, et al. Strategies for retention of heterosexual men in HIV care in sub-Saharan Africa: A systematic review. PloS One. 2021 Feb 4;16(2):e0246471. doi: 10.1371/journal.pone.0246471 33539424 PMC7861356

[43] Loubiere S, Peretti-Watel P, Boyer S, Blanche J, Abega S-C, Spire B. HIV disclosure and unsafe sex among HIV-infected women in Cameroon: results from the ANRS-EVAL study. Soc Sci Med. 2009 Sep 1;69(6):885–91. doi: 10.1016/j.socscimed.2009.05.044 19560244

[44] Tshweneagae GT, Oss VM, Mgutshini T. Disclosure of HIV status to sexual partners by people living with HIV. Curationis. 2015 Jan 1;38(1):1–6. doi: 10.4102/curationis.v38i1.1174 26017276 PMC6091570

[45] Obiri-Yeboah D, Amoako-Sakyi D, Baidoo I, Adu-Oppong A, Rheinländer T. The ‘Fears’ of Disclosing HIV Status to Sexual Partners: A Mixed Methods Study in a Counseling Setting in Ghana. AIDS Behav. 2016 Jan;20:126–36. doi: 10.1007/s10461-015-1022-1 25711298

[46] Shushtari ZJ, Sajjadi H, Forouzan AS, Salimi Y, Dejman M. Disclosure of HIV status and social support among people living with HIV. Iranian Red Crescent Medical Journal. 2014 Aug;16(8). 10.5812/ircmj.11856 PMC4221996 25389470

[47] Smith R, Rossetto K, Peterson BL. A meta-analysis of disclosure of one’s HIV-positive status, stigma and social support. AIDS care. 2008 Nov 1;20(10):1266–75. doi: 10.1080/09540120801926977 18608080

[48] Gielen AC, Fogarty L, O’Campo P, Anderson J, Keller J, Faden R. Women living with HIV: disclosure, violence, and social support. J Urban Health Bull. 2000 Sep;77:480–91. doi: 10.1007/BF02386755 10976619 PMC3456042

[49] Nelson LE, Wilton L, Agyarko‐Poku T, Zhang N, Aluoch M, Thach CT, et al. The association of HIV stigma and HIV/STD knowledge with sexual risk behaviors among adolescent and adult men who have sex with men in Ghana, West Africa. Research in nursing & health. 2015 Jun;38(3):194–206. doi: 10.1002/nur.21650 25809638

[50] Thirumurthy H, Jakubowski A, Camlin C, Kabami J, Ssemmondo E, Elly A, et al. Expectations about future health and longevity in Kenyan and Ugandan communities receiving a universal test-and-treat intervention in the SEARCH trial. AIDS Care. 2016 Jun 2;28(Suppl 3):90–8. doi: 10.1080/09540121.2016.1178959 27421056 PMC5443252

[51] Dias S, Gama A, Loos J, Roxo L, Simões D, Nöstlinger C. The role of mobility in sexual risk behaviour and HIV acquisition among sub-Saharan African migrants residing in two European cities. PLoS One. 2020 Feb 5;15(2):e0228584. doi: 10.1371/journal.pone.0228584 32023309 PMC7001961

