# Supplementary material for: Sex specific differences in HIV status disclosure and care engagement among people living with HIV in rural communities in Kenya and Uganda
Source: PLOS Glob Public Health. 2023 Apr 7;3(4):e0000556. doi: 10.1371/journal.pgph.0000556 (PMC10081749; doi:10.1371/journal.pgph.0000556)
Supplement: Attachment — Submitted filename: Responses to reviewers.docx [file pgph.0000556.s002.docx]

**Review Comments to the Author**

**Reviewer #1: Specific Comments**

Abstract part

• The abstract part has been developed in somewhat attractive way, but if it includes the aim of the study it will provide some clue to reader about what it is without needing of visiting the research topic.

Response: Thank you for this comment. We have updated to abstract to include the aim of the study. The abstract now includes the following sentence, “We sought to explore experiences with and correlates of disclosure among PLHIV participating in a study of population mobility.”

• It concluded that there is a gender difference on disclosing, care and condom use, but as a recommendation it puts as follow “Interventions focused on differing disclosure support needs for women and men are needed”. So, if there is a gender difference, why you have to recommend for the specific gender with a gap?

Response: Thank you for this comment. We have considered your point and updated the final sentence of the abstract to read, “Interventions focused on differing disclosure support needs for women and men are needed and may help facilitate better care engagement for men and women and improve condom use in men.”

• The following sentence is duplicated “Unmarried versus married men had higher odds of non- disclosure (aOR=4.65, 95%CI, 1.32-16.35) and no condom use in the past 6 months (aOR=4.80, 95%CI, 1.74-13.20) and lower odds of receiving HIV care (aOR=0.15; 95%CI, 0.04- 50 0.49).” so revise it.

Response: Thank you for this comment. The sentence highlighted was not a duplicate. The first sentence was about unmarried versus married MEN, while the second sentence was about unmarried versus married WOMEN. However, we have updated the sentence for clarity. It now reads, “Unmarried versus married men had higher odds of non- disclosure (aOR=4.65, 95%CI, 1.32-16.35) and no condom use in the past 6 months (aOR=4.80, 95%CI, 1.74-13.20), as well as lower odds of receiving HIV care (aOR=0.15; 95%CI, 0.04- 50 0.49).”

Methods and material part

• It needs an operational definition for HIV status disclosure

Response: Thank you for this comment. We have added a definition of disclosure in the text. It now reads, “HIV status disclosure was defined as PLHIV who reported voluntary disclosure of their HIV-positive status to at least one person”.

Result part

• Some of the findings are mentioned both in text and in table, so if the response of majority has been mentioned in text then the remain can be described in the table, otherwise it would be a redundancy to the reader. (for instance some the information which are included at table 2 and 3 are already described in text form above the tables)

Response: Thank you for this comment. We have edited the text to reduce redundancy in the results.

Discussion part

• I suggest the first paragraph may not be necessary, because it has described at the consecutive paragraph while the specific variable being discussed

Response: Thank you for this comment. We have retained some of the first paragraph of the discussion, but have removed some sections that were restated in later discussion paragraphs.

Thank you, Reviewer 1 for your helpful comments.

**Reviewer #2:** In the manuscript entitled ‘Sex specific differences in HIV status disclosure and care engagement among people living with HIV in rural communities in Kenya and Uganda’ Okorie et al., have investigated the impact of sex-specific pattern of HIV disclosure among a cohort of PLHIV in Rural Kenya and Uganda. The study documented the sex-specific challenges faced by PLHIV on the issue of HIV disclosure. The important highlight of this study lies in the gender differences in barriers to HIV disclosure, use of condoms and engagement in HIV care bringing to the fore a need for providing different emotional and organizational support for men and women. It is an interesting study conducted with standard methodologies. However, the following comments need to be addressed:

1. Under the introduction section, p.3, line 76, kindly replace “those who choose not to disclose, non-disclosure is a way to” to “those who choose not to disclose, it is a way to”

Response: Thank you for this comment. The text has been adjusted as requested to read, “For those who choose not to disclose, it is a way to protect their identity and avoid stigma and discrimination while maintaining a sense of self and safety.”

2. P.3, line 79, please change “study focuses on voluntary disclosure (fully disclosing, selective disclosing, or non-disclosure” to “study focuses on voluntary disclosure (full, selective, or non-disclosure”

Response: Thank you for this comment. The text has been adjusted as requested to read, “However, this study focuses on voluntary disclosure (full, selective, or non-disclosure)”.

3. In the methods section, under data collection, the authors did not mention translating from the local languages used for administering the surveys into English language. I suggest that the authors consider doing so for the sake of clarity.

Response: The text has been adjusted as requested and now reads, “Surveys were originally developed in English and then translated and administered (using portable tablets) in the local languages (Lusoga, Lugwere, Ateso, Runyankole, and Dholuo) of the research participants, following procedures for the protection of privacy and confidentiality.”

4. P.15, line 314: please change “When stratified by sex, we see” to “When stratified by sex, we found”

Response: Text has been adjusted as requested and now reads, “When stratified by sex, we found that men who have never disclosed have higher odds of being unmarried and reporting no condom-use in the past 6-months and lower odds of HIV care engagement, while women who had never disclosed their HIV status had higher odds of being unmarried and lower odds of HIV care engagement.”

Thank you, Reviewer 2 for your helpful comments.

**Reviewer #3:** In Abstract, may change from ‘Non-disclosure was associated with no past 6-month condom use’ to ‘Non-disclosure was associated with no condom use in past 6 months’ to keep terminology consistent with no condom use and make it less confusing.

Response: Thank you for this comment. The text has been adjusted as requested and now reads, “Non-disclosure was associated with no condom use in the past 6 months (aOR=2.44; 95%CI, 1.40-4.25) and with lower odds of receiving care (aOR=0.8; 95%CI, 0.04-0.17).”

Add ‘can’ in following sentence. Otherwise, it infers that non-disclosure always leads to negative outcomes:

- Alternatively, non-disclosure, which is often a manifestation of HIV-related stigma (whether internalized, anticipated, or in response to enacted stigma), ‘can’ negatively impacts care outcomes.

Response: All text has been adjusted as requested and now reads, “Alternatively, non-disclosure, which is often a manifestation of HIV-related stigma (whether internalized, anticipated, or in response to enacted stigma), can negatively impact care outcomes.”

- Not sure what the following sentence means: ‘Even with high disclosure rates, disclosing is not without its emotional and relational consequences’.

Response: Thank you for this comment. We have updated the text for clarity. It now reads, “Even with high disclosure rates in this study**,** disclosing was not without its emotional, relational, and psychological hardships.”

Will be good to discuss possible causes of why non-disclosure is associated with no condom use in the discussion section.

Response: Thank you for this suggestions. We have added a discussion about possible causes for why non-disclosure is associated with no condom use in men. The text now reads, “In addition, men who had not disclosed were not using condoms. This effect was not seen among women. It is possible that men feared that using condoms might signal their HIV-positive status to a partner or lead to stigma, as prior literature among men who have sex with men has suggested. Research suggests that sometimes men have used avoidant disclosure strategies (i.e. introduction of condom use) under the pretext of family planning and protecting their partners while women have used such techniques when they were unsure of their partner’s HIV status [2]. it is usually easier for men living with HIV than women to disclose based on gender differences in sexual decision-making power [2]”.

Thank you, Reviewer 3 for your feedback. We have made all revisions as suggested.

**General comments**

1. Write out the full meaning of every abbreviation at the first instance including those used in the abstract e.g., HIV.

Response: We have revised to manuscript to ensure that all abbreviations have been defined when they first appear in the text.

2. Can you include the justifications for included characteristics of adults who had and had not disclosed HIV-positive status?

Response: Thank you for this comment. The goal of this paper was to examine sex differences in patterns of disclosure among people living with HIV, regardless of whether or not they had disclosed their status. Including the characteristics of PLHIV who had disclosed and had not non-disclosed helps to provide a complete picture of HIV status disclosure in the study population.

We have now included a justification for the characteristics that have been included in models. The text now reads, “Pooled, and sex-stratified mixed effects logistic regression models were fitted to examine associations of HIV-positive status non-disclosure with sexual risk behaviors including past 6-month condom use, past year relationship concurrency, and HIV care engagement controlling for age, marital status, region and adjusted for clustering at the community-level. These factors were included in models because prior research has suggested an association between disclosure and age and marital status [37–39].”

3. Blurry figures. Upload better and clearer figures

Response: The figure has been adjusted as requested.

**Other General comments and recommendations**

Edited the Financial Disclosure to reflect only what the editors requested.

This research was supported by the National Institutes of Health, NIMH (R01MH104132 to CS), and the National Institutes of Mental Health of the U.S. Public Health Service (T32 MH19105 to SAG). The study's funders had no role in study design, data collection, data analysis, data interpretation, or writing the report.

Supplementary Document: S1 Supporting information is added to the manuscript intext and stated as a list of captions at the end of the manuscript. The inclusivity document was relabeled as S1 Supporting information and uploaded to PLOS.

Created repository to adhere to PLOS open data policy. The repository is published at

Gutin, Sarah; Okorie, Chinomnso (2022), Survey: HIV status disclosure & care engagement in a ‘test and treat’ trial population, Kenya & Uganda, 2022, Dryad, Dataset, <https://doi.org/10.7272/Q69P2ZWR>
