# Supplementary material for: Sex specific differences in HIV status disclosure and care engagement among people living with HIV in rural communities in Kenya and Uganda
Source: PLOS Glob Public Health. 2023 Apr 7;3(4):e0000556. doi: 10.1371/journal.pgph.0000556 (PMC10081749; doi:10.1371/journal.pgph.0000556)
Supplement: Attachment — Submitted filename: Responses to reviewers 2.3.23.docx [file pgph.0000556.s003.docx]

**Review Comments to the Author**

Reviewers' comments:

**Reviewer #4:**

1. If the authors have adequately addressed your comments raised in a previous round of review and you feel that this manuscript is now acceptable for publication, you may indicate that here to bypass the “Comments to the Author” section, enter your conflict of interest statement in the “Confidential to Editor” section, and submit your "Accept" recommendation.

Reviewer #4: All comments have been addressed

Response: Thank you for this comment. We are glad that address all comments stated by previous reviewers.

2. Does this manuscript meet PLOS Global Public Health’s publication criteria? Is the manuscript technically sound, and do the data support the conclusions? The manuscript must describe methodologically and ethically rigorous research with conclusions that are appropriately drawn based on the data presented.

Reviewer #4: Yes

Response: Thank you for this comment.

3. Has the statistical analysis been performed appropriately and rigorously?

Reviewer #4: Yes

Response: Thank you for this comment.

4. Have the authors made all data underlying the findings in their manuscript fully available (please refer to the Data Availability Statement at the start of the manuscript PDF file)?

The PLOS Data policy requires authors to make all data underlying the findings described in their manuscript fully available without restriction, with rare exception. The data should be provided as part of the manuscript or its supporting information, or deposited to a public repository. For example, in addition to summary statistics, the data points behind means, medians and variance measures should be available. If there are restrictions on publicly sharing data—e.g. participant privacy or use of data from a third party—those must be specified.

Reviewer #4: Yes

Response: Thank you for this comment.

5. Is the manuscript presented in an intelligible fashion and written in standard English?

PLOS Global Public Health does not copyedit accepted manuscripts, so the language in submitted articles must be clear, correct, and unambiguous. Any typographical or grammatical errors should be corrected at revision, so please note any specific errors here.

Reviewer #4: Yes

Response: Thank you for this comment.

6. Review Comments to the Author

Please use the space provided to explain your answers to the questions above. You may also include additional comments for the author, including concerns about dual publication, research ethics, or publication ethics. (Please upload your review as an attachment if it exceeds 20,000 characters)

Reviewer #4: (No Response)

7. PLOS authors have the option to publish the peer review history of their article (what does this mean?). If published, this will include your full peer review and any attached files.

Do you want your identity to be public for this peer review? If you choose “no”, your identity will remain anonymous but your review may still be made public.

For information about this choice, including consent withdrawal, please see our Privacy Policy.

Reviewer #4: Yes: Dr Sheriff Abdulrahman

Response: Thank you for your time reviewing our paper.

Journal Requirements:

Please review your reference list to ensure that it is complete and correct. If you have cited papers that have been retracted, please include the rationale for doing so in the manuscript text, or remove these references and replace them with relevant current references. Any changes to the reference list should be mentioned in the rebuttal letter that accompanies your revised manuscript. If you need to cite a retracted article, indicate the article’s retracted status in the References list and also include a citation and full reference for the retraction notice.

Response: Thank you for your feedback. We have reviewed and updated the reference list using the PLOS Global Public Health guidelines.

Additional Editor Comments (if provided):

Good revision but need to make the figures clearer.

Response: Thank you for your feedback. We updated our image resolution and checked the images using the Preflight Analysis and Conversion Engine (PACE) digital diagnostic tool as requested.
